# Prevalence of the Metabolic Syndrome among Korean Workers by Occupational Group: Fifth Korean National Health and Nutrition Examination Survey (KNHANES) 2010

**DOI:** 10.1186/2052-4374-25-13

**Published:** 2013-08-05

**Authors:** Ji Young Ryu, Sukwoo Hong, Chang-Hyo Kim, Sangyoon Lee, Jeong-Ho Kim, Jong-Tae Lee, Dae Hwan Kim

**Affiliations:** 1 Department of Occupational and Environmental Medicine, Inje University Haeundae Paik Hospital, 875 Heaundae-ro, Haeundae-Gu, Busan 612-862, South Korea; 2 Inje University Graduate School, 197 Inje-ro, Gimhae, Gyungnam, South Korea; 3 Department of Occupational and Environmental Medicine, College of Medicine & Institute of Environmental and Occupational Medicine, Inje University, 75 Bokji-ro Busanjin-gu, Busan, South Korea

**Keywords:** Metabolic syndrome, Prevalence, Workers, Occupation

## Abstract

**Objectives:**

The prevalence of the metabolic syndrome has increased rapidly in South Korea over the past 10 years. However, the occurrence of the metabolic syndrome in workers grouped according to the specific type of work is not well understood in Korea. In this study, we assessed the differences in the prevalence of the metabolic syndrome by occupational group and evaluated the risk of the metabolic syndrome among occupational groups.

**Methods:**

From the Fifth Korean National Health and Nutrition Examination Survey (2010), 3,303 employed participants were included in this study. The unadjusted and age-adjusted prevalences of the metabolic syndrome were estimated and multiple logistic regression analysis was conducted using the presence of the metabolic syndrome as a dependent variable, and adjusting for age, education level, household income, drinking behavior, smoking status, physical activity, work hours, and work scheduling pattern.

**Results:**

Among male workers, non-manual workers had the greatest age-adjusted prevalence (26.4%, 95% CI: 22.3-30.5%) among the occupational groups. In a logistic regression analysis, male manual workers had a significantly lower odds ratio for the metabolic syndrome relative to non-manual workers (0.59, 95% CI: 0.41-0.85).

**Conclusion:**

Our study demonstrated differences in the prevalence of the metabolic syndrome by occupational group and identified the greatest risk for the metabolic syndrome in male non-manual workers.

## Introduction

The metabolic syndrome is a condition that is defined by the combination of five factors: abdominal obesity, hypertension, hypertriglyceridemia, low HDL-cholesterol level, and hyperglycemia [1]. These components of the metabolic syndrome are major risk factors for cardiovascular disease and impaired glucose metabolism [1-4].

Recently, the prevalence of the metabolic syndrome has increased rapidly in South Korea [5,6]. According to a study using the data from the Korean National Health and Nutrition Examination Survey (KNHANES), the age-adjusted prevalence of the metabolic syndrome in 2007 was 31.3%, while the age-adjusted prevalence of the metabolic syndrome in 1998 had been just 24.9% [6]. It is thought that the rapid increase in the prevalence of the metabolic syndrome may have arisen from lifestyle changes including a westernized diet and physical inactivity [5-7].

On the other hand, recent studies have shown that the prevalence of the metabolic syndrome varies in different occupational groups [8,9]. Among U.S. workers, the unadjusted and age-adjusted prevalences of the metabolic syndrome were greatest in “transportation and material occupations” and “food preparation and food service workers,” respectively [9]. In male Spanish workers, the highest age-adjusted prevalence of the metabolic syndrome was found among “machine installers, operators and assemblers” while the prevalence of the metabolic syndrome in female workers was highest among “skilled workers in the agricultural and fishing industries” [8]. It is expected that the prevalence of the metabolic syndrome among workers may differ by country, considering the distinctive working environment of each country. In Korea, only a few studies have assessed the association between the occupational group and the metabolic syndrome. Although previous research by Myoung et al. has evaluated the relationship between occupations and the metabolic syndrome among Korean workers, the study was limited by the fact that the occupations were grouped into manual or non-manual workers only, without considering service and sales workers as a separate work group [10].

In this study, we assessed the differences in the prevalence of the metabolic syndrome and its individual criteria by occupational group and evaluated the risk of the metabolic syndrome among occupational groups using KNHANES data.

## Materials and methods

### Subjects

Data from the fifth KNHANES, gathered in 2010, was used for the analyses. The KNHANES is a multistage stratified complex design survey of a representative sample of the entire Korean population conducted by the Korea Centers for Disease Control and Prevention. The total number of participants was 8,958, and the participation rate was 81.9%. Trained interviewers and laboratory technicians conducted surveys in households, including administering questionnaires, performing health examinations, and collecting blood samples. Subjects who were at least 20 years old, who reported having jobs, and who had occupational information were included in this study (n = 3,667). Subjects who reported having no jobs (n = 4,595), who had no occupational information (n = 597), or who belonged to the armed forces group in the occupational classification (n = 31) were excluded from this study. After this exclusion, the workers who were aged below 20 years old were also excluded (n = 68). Among 3,667 included workers, 3,303 workers who had fully available data needed for diagnosis of the metabolic syndrome were analyzed. This study was approved by the Institutional Review Board of the Haeundae Paik Hospital.

### Occupational classification

The subjects were classified into nine occupational subgroups based on the KNHANES data. The KNHANES employed the major categories of the Korean Standard Classification of Occupations (KSCO) [11] as the occupational classification. These occupations were grouped into three categories according to their degree of physical work: non-manual workers, service/sales workers, and manual workers. Non-manual workers were managers, professionals and related workers, and clerks. Service workers and sales workers constituted one distinctive group. Manual workers consisted of skilled agricultural, forestry and fishery workers, craft and related trades workers, equipment, machine operating and assembling workers, and elementary workers.

### Definition of the metabolic syndrome

The presence of the metabolic syndrome was determined by the consensus definition [1] incorporating International Diabetes Federation (IDF) worldwide criteria [12] and American Heart Association/National Heart, Lung, and Blood Institute (AHA/NHLBI) criteria [13]. An individual who had any three or more of the following five criteria was defined as having the metabolic syndrome: 1) waist circumference ≥ 90 cm in men and ≥ 80 cm in women, 2) blood pressure ≥ 130/85 mmHg or receiving anti-hypertensive medication, 3) triglycerides ≥ 150 mg/dl, 4) HDL-cholesterol < 40 mg/dl in men and < 50 mg/dl in women, 5) fasting glucose ≥ 100 mg/dl or taking blood glucose lowering drugs.

### Covariates

Age, level of education, household income, smoking status, high-risk drinking behavior, vigorous physical activity, work hours, work scheduling pattern, and body mass index (BMI) were included as covariates in this study. The levels of education were categorized into elementary school graduates (6-year course), middle school graduates (3-year), high school graduates (3-year), and some university education or above (2 or more years). Household income was classified by quartile as low, low-middle, middle-high, or high levels. Smoking status was divided into non-smokers, ex-smokers, and current smokers. High-risk drinking was defined as at least 7 glasses per day in men and at least 5 glasses per day in women and drinking behavior was categorized into no, once a month, once a week, or every day experience of high-risk drinking. Vigorous physical activity was defined as physical activities over 10 minutes that are more strenuous than usual activities or make one breathless, including carrying heavy loads, running, climbing, fast cycling, fast swimming, soccer, basketball, and so on. This variable was divided into none, below 5 days per week, and at least 5 days per week of vigorous physical activities. Work hours were categorized into 40 hours per week or less and over 40 hours per week. Work schedule patterns were grouped as either daytime fixed work or non-daytime work, including night-time and shift work. BMI was classified into normal/underweight (below 25 kg/m^2^), overweight (between 25 and 30 kg/m^2^), and obese (more than 30 kg/m^2^).

### Statistical analyses

The statistical analyses were performed using SAS Enterprise Guide (version 4.2; SAS Institute Inc., Cary, NC) to take into account sample weights and complex sample design effects. All of the data are presented as an estimated percentage (standard error) or estimated mean (standard error) for the demographic variables and each component of the metabolic syndrome. The Rao-Scott chi-squared test was used to compare the prevalence of the categorical variables. The unadjusted and age-adjusted prevalences of the metabolic syndrome for nine occupational groups were estimated. Age-standardization by the direct method was done for the prevalence estimates using 2010 Census population data of Korea. The prevalence estimate for each occupational group was considered significantly higher than that for workers overall if the estimate was above the upper bounds of the 95% confidence interval for the overall prevalence estimate [14]. For the occupational groups, multiple logistic regression analysis was conducted with the presence of the metabolic syndrome as a dependent variable after stratification for gender. The multiple logistic regression analysis was performed, adjusting for age (continuous variable), level of education, household income, drinking behavior, smoking status, physical activity, work hours, and work schedule pattern.

## Results

Tables 1 and 2 present the demographic characteristics by the major job categories of the KSCO among male and female workers, respectively. In both the male and female workers, “managers”, “professional and related” workers, and “clerks” had high levels of education and household income. Among the male workers, the proportion doing vigorous physical activity at least 5 days per week was lowest in the “managers” (3.5%), followed by “professional and related” workers (3.8%) and “clerks” (5.8%) and highest in the “skilled agricultural, forestry and fishery workers” (17.5%), followed by “elementary” workers (14.8%), sales workers (12.7%), and service workers (12.3%). The proportion of those with less than 40 work hours per week was higher in the “managers” (48.4%), “professional and related” workers (41%), and “clerks” (42.6%) than in the other work groups in the men. Service workers reported the highest proportion of non-day work in both the male (51.4%) and female (29.8%) workers. “Managers”, “professional and related” workers, and “clerks” were more likely to be overweight or obese than those of the other occupational groups in the male workers. On the other hand, in female workers, the proportion of overweight or obese individuals was smaller among the “managers”, “professional and related” workers, and “clerks” than the other occupational groups.

**Table 1:** Demographic characteristics by major category of the Korean Standard Classification of Occupations among male workers

	**Managers**	**Professional and related workers**	**Clerks**	**Service workers**	**Sales workers**	**Skilled agricultural, forestry and fishery workers**	**Craft and related trades workers**	**Equipment, machine operation and assembling workers**	**Elementary workers**	**Total**	**p-value**
Age (years)*	50.9 (1.3)	38.2 (0.7)	39.1(0.7)	36.8 (1.4)	41.2 (1.2)	56.4 (1.4)	42.7 (0.9)	45.1 (1.1)	46.1 (1.7)	44.1 (0.4)	< 0.001
Education											< 0.001
≤ Elementary school	0.0 (0.0)	0.6 (0.4)	0.4 (0.4)	4.9 (2.1)	6.5 (2.1)	41.5 (3.3)	14.2 (2.7)	7.7 (1.8)	21.7 (4.3)	10.7 (1.1)	
Middle school	16.3 (5.6)	0.4 (0.3)	1.7 (1.0)	6.4 (2.4)	7.6 (2.2)	20.8 (3.9)	13.9 (2.5)	19.7 (3.0)	17.5 (3.6)	10.3 (0.9)	
High school	31.8 (6.7)	21.0 (3.6)	27.6 (3.4)	56.3 (5.4)	45.7 (4.4)	24.9 (3.2)	53.3 (4.0)	56.9 (4.0)	50.5 (4.9)	39.2 (1.5)	
≥ University	51.8 (6.7)	78.0 (3.6)	70.3 (3.6)	32.4 (4.8)	40.2 (3.9)	12.8 (2.8)	18.6 (3.0)	15.7 (3.2)	10.3 (2.8)	39.8 (1.7)	
Household income											< 0.001
Low	3.7 (2.7)	6.7 (1.8)	2.7 (1.1)	6.5 (2.7)	9.3 (2.2)	24.1 (4.0)	12.3 (2.8)	8.6 (2.4)	26.5 (4.2)	10.9 (1.0)	
Low-middle	10.6 (4.4)	17.1 (2.7)	24.5 (2.8)	34.6 (2.7)	27.6 (3.6)	28.2 (4.3)	35.4 (3.7)	26.9 (3.5)	29.2 (4.3)	26.4 (1.4)	
Middle-high	29.2 (7.2)	39.0 (3.0)	33.9 (3.1)	25.0 (4.4)	36.1 (4.3)	18.2 (2.6)	29.0 (3.4)	37.6 (3.6)	29.5 (4.9)	31.9 (1.4)	
High	56.5 (6.1)	37.3 (2.9)	38.9 (3.7)	33.9 (5.2)	27.0 (3.7)	29.5 (4.2)	23.4 (3.3)	26.9 (3.4)	14.8 (3.6)	30.7 (1.6)	
High-risk drinking											0.002
Never	17.5 (5.2)	12.7 (2.1)	14.1 (2.3)	7.4 (2.6)	13.0 (2.7)	18.1 (3.6)	14.1 (2.5)	14.8 (2.3)	26.4 (4.6)	14.8 (1.0)	
Once a month	13.4 (6.3)	46.3 (3.4)	38.9 (3.3)	44.4 (5.8)	33.7 (4.0)	32.6 (4.3)	42.5 (4.5)	39.2 (4.3)	35.9 (5.5)	40.0 (1.5)	
Once a weak	30.0 (6.6)	30.2 (3.2)	38.3 (3.6)	35.5 (5.3)	43.6 (4.3)	26.2 (4.1)	28.8 (3.8)	29.7 (3.5)	20.2 (4.7)	31.8 (1.2)	
Everyday	9.1 (4.2)	10.7 (2.0)	8.7 (2.2)	12.7 (3.6)	9.8 (2.6)	23.2 (3.3)	14.6 (2.7)	16.3 (3.2)	17.5 (4.0)	13.4 (1.1)	
Smoking status											0.027
Non-smoker	15.0 (4.4)	23.7 (2.4)	17.7 (2.6)	18.7 (3.9)	17.9 (3.1)	13.9 (2.8)	12.0 (2.4)	10.9 (2.5)	15.5 (3.9)	16.5 (1.0)	
Ex-smoker	41.1 (6.1)	30.1 (2.9)	31.6 (2.8)	23.9 (4.4)	31.8 (3.7)	41.8 (3.0)	32.7 (3.6)	34.4 (3.1)	29.5 (4.3)	32.6 (1.3)	
Current smoker	43.9 (6.0)	46.3 (3.4)	50.8 (2.9)	57.4 (5.1)	50.3 (4.4)	44.3 (3.5)	55.4 (3.7)	54.7 (3.4)	55.0 (5.3)	50.9 (1.4)	
Vigorous physical activity											< 0.001
None	66.2 (6.4)	53.3 (3.8)	44.4 (3.6)	52.1 (5.1)	50.2 (3.5)	60.7 (3.3)	53.3 (4.2)	49.4 (3.7)	58.8 (5.2)	52.7 (1.4)	
< 5 days per week	30.3 (6.1)	42.8 (3.9)	49.7 (3.6)	35.7 (5.2)	37.1 (4.1)	21.8 (2.8)	36.1 (4.1)	41.4 (3.7)	26.4 (5.2)	37.6 (1.4)	
≥ 5 days per week	3.5 (2.1)	3.8 (1.2)	5.8 (1.4)	12.3 (3.6)	12.7 (3.0)	17.5 (2.6)	10.7 (2.2)	9.2 (2.4)	14.8 (4.1)	9.7(0.8)	
Work hours											< 0.001
≤ 40 hours per week	48.8 (6.5)	41.0 (3.6)	42.6 (3.3)	28.5 (4.7)	28.3 (3.9)	37.8 (4.4)	25.6 (2.9)	23.6 (3.2)	39.4 (4.8)	34.4 (1.3)	
> 40 hours per week	51.2 (6.5)	59.0 (3.6)	57.4 (3.3)	71.5 (4.7)	71.7 (3.9)	62.2 (4.4)	74.4 (2.9)	76.4 (3.2)	60.6 (4.8)	65.6 (1.3)	
Work schedule pattern											< 0.001
Day	92.0 (4.5)	81.7 (2.5)	90.9 (2.2)	48.6 (5.7)	84.8 (3.0)	99.8 (0.2)	87.2 (3.5)	72.9 (3.3)	68.5 (4.1)	82.2 (1.2)	
Night/overnight	3.7 (2.0)	13.6 (2.3)	2.6 (1.1)	26.3 (5.3)	14.2 (3.0)	0.1 (0.1)	2.0 (1.3)	8.7 (2.0)	11.3 (2.8)	8.6 (0.8)	
Shift	0.0	1.5 (0.9)	5.2 (1.9)	15.5 (4.1)	0.3 (0.2)	0.0	8.4 (2.7)	11.3 (2.5)	19.4 (3.2)	6.3 (0.8)	
Others	4.2 (4.1)	3.2 (1.0)	1.3 (0.7)	9.6 (2.7)	0.7 (0.5)	0.1 (0.1)	2.4 (2.0)	7.1 (2.2)	0.8 (0.7)	2.9 (0.5)	
BMI category											0.230
Normal/underweight	55.2 (7.6)	56.6 (3.0)	58.1 (3.5)	60.5 (5.8)	62.8 (4.2)	68.5 (3.5)	61.4 (3.3)	62.2 (3.7)	67.0 (4.5)	61.3 (1.4)	
Overweight	37.5 (6.4)	39.3 (2.9)	36.4 (3.6)	37.7 (5.7)	30.5 (3.7)	30.8 (3.4)	35.2 (3.2)	33.5 (3.6)	30.1 (4.5)	34.7 (1.3)	
Obese	7.3 (3.7)	4.1 (1.3)	5.5 (1.7)	1.8 (1.3)	6.7 (2.0)	0.7 (0.5)	3.4 (1.5)	4.3 (1.5)	2.9 (1.4)	3.9 (0.6)	

*Estimated mean (standard error).

**Table 2:** Demographic characteristics by major category of the Korean Standard Classification of Occupations among female workers

	**Managers**	**Professional and related workers**	**Clerks**	**Service workers**	**Sales workers**	**Skilled agricultural, forestry and fishery workers**	**Craft and related trades workers**	**Equipment, machine operation and assembling workers**	**Elementary workers**	**Total**	**p-value**
Age (yr)*	54.1 (3.9)	34.2 (0.6)	33.7 (0.7)	43.2 (1.0)	43.2 (1.0)	57.4 (1.5)	49.1 (2.9)	41.8 (2.4)	51.7 (1.0)	45.4 (0.6)	< 0.001
Education											< 0.001
≤ Elementary school	0.0 (0.0)	0.6 (0.3)	0.3 (0.3)	21.7 (3.3)	18.4 (2.6)	67.2 (4.7)	30.4 (9.7)	16.5 (7.4)	39.8 (3.0)	22.3 (1.9)	
Middle school	18.2 (15.1)	0.6 (0.4)	2.2 (1.0)	18.3 (2.9)	13.3 (2.5)	15.5 (4.5)	34.5 (8.4)	17.2 (7.6)	18.5 (3.4)	11.6 (1.1)	
High school	18.4 (12.9)	17.1 (2.5)	50.1 (3.9)	47.3 (3.6)	47.6 (3.7)	15.0 (4.2)	31.8 (7.6)	60.4 (10.3)	34.7 (3.7)	34.5 (1.7)	
≥ University	63.3 (18.0)	81.7 (2.5)	47.4 (4.1)	12.6 (2.7)	20.7 (3.0)	2.3 (1.4)	3.3 (2.3)	5.8 (4.1)	6.9 (1.8)	31.5 (1.8)	
Household income											< 0.001
Low	0.0 (0.0)	4.0 (1.4)	2.6 (1.2)	10.0 (2.3)	12.1 (2.3)	35.7 (4.9)	30.6 (9.4)	0.0 (0.0)	25.1 (2.9)	14.2 (1.3)	
Low-middle	1.2 (1.2)	17.2 (2.7)	20.7 (3.8)	31.1 (3.6)	23.0 (3.8)	25.6 (3.2)	23.6 (6.1)	23.9 (7.7)	36.9 (3.4)	25.4 (1.5)	
Middle-high	14.6 (13.3)	37.4 (3.3)	36.8 (4.2)	30.7 (3.8)	35.4 (3.8)	18.0 (3.3)	28.2 (7.6)	45.2 (10.9)	22.6 (2.5)	30.8 (1.5)	
High	84.2 (13.4)	41.4 (3.4)	40.0 (3.8)	28.2 (3.3)	29.6 (3.7)	20.7 (3.2)	17.6 (6.5)	30.9 (9.5)	15.4 (2.6)	29.6 (1.6)	
High-risk drinking											< 0.001
Never	77.4 (15.6)	39.6 (3.5)	31.7 (3.9)	35.9 (3.9)	43.6 (3.8)	57.0 (5.4)	18.5 (6.7)	37.1 (11.3)	42.7 (5.0)	39.8 (1.7)	
Once a month	22.6 (15.6)	45.0 (3.7)	52.9 (4.3)	43.5 (4.0)	37.8 (4.2)	38.4 (4.4)	58.6 (9.3)	59.8 (11.7)	35.9 (3.9)	43.6 (4.6)	
Once a weak	0.0 (0.0)	15.4 (3.2)	14.7 (3.0)	13.6 (2.6)	17.2 (3.3)	4.0 (2.2)	19.5 (8.5)	3.1 (3.1)	14.3 (3.3)	13.8 (1.3)	
Everyday	0.0 (0.0)	0.0 (0.0)	0.7 (0.7)	7.0 (2.0)	1.3 (1.3)	0.6 (0.6)	3.4 (2.6)	0.0 (0.0)	7.1 (3.0)	2.7 (0.6)	
Smoking status											0.015
Non-smoker	100.0 (0.0)	88.5 (1.9)	86.3 (2.7)	86.0 (3.1)	83.4 (3.0)	96.4 (1.7)	91.8 (5.6)	96.7 (3.3)	83.0 (3.2)	87.4 (1.2)	
Ex-smoker	0.0 (0.0)	7.5 (1.6)	7.9 (2.2)	4.9 (1.7)	9.5 (2.4)	2.3 (1.4)	8.0 (5.6)	0.0 (0.0)	5.4 (1.7)	6.3 (0.8)	
Current smoker	0.0 (0.0)	4.0 (1.1)	5.8 (1.8)	9.2 (2.9)	7.1 (2.2)	1.3 (0.8)	0.1 (0.1)	3.3 (3.3)	11.6 (3.1)	6.3 (0.9)	
Vigorous physical activity											0.007
None	55.8 (18.7)	74.1 (3.1)	70.2 (3.7)	70.1 (3.7)	69.5 (3.8)	76.1 (4.1)	86.3 (5.4)	61.5 (8.8)	75.0 (3.2)	72.9 (1.4)	
< 5 days per week	44.2 (18.7)	21.8 (2.9)	26.2 (3.6)	20.4 (3.6)	23.7 (3.3)	11.4 (2.7)	6.5 (3.6)	23.9 (8.4)	16.2 (2.4)	19.8 (1.2)	
≥ 5 days per week	0.0 (0.0)	4.1 (1.3)	3.6 (1.4)	9.5 (2.3)	6.8 (2.0)	12.5 (3.3)	7.2 (4.3)	14.6 (5.6)	8.8 (2.0)	7.3 (0.8)	
Work hours											< 0.001
≤ 40 hours per week	80.4 (13.9)	57.0 (3.5)	52.4 (4.2)	33.7 (4.0)	48.9 (4.0)	50.9 (4.0)	29.4 (7.3)	35.9 (9.9)	64.5 (3.6)	51.2 (1.6)	
> 40 hours per week	19.6 (13.9)	43.0 (3.5)	47.6 (4.2)	66.3 (4.0)	51.1 (4.0)	49.1 (4.0)	70.6 (7.3)	64.1 (9.9)	35.5 (3.6)	48.8 (1.6)	
Work schedule pattern											<0.001
Day	93.8 (5.4)	74.8 (3.2)	94.3 (2.0)	70.2 (3.7)	77.7 (3.4)	99.0 (0.7)	93.0 (4.6)	74.2 (9.6)	85.8 (2.7)	82.7 (1.5)	
Night/overnight	5.1 (5.1)	18.9 (3.0)	3.2 (1.5)	24.5 (3.4)	15.6 (2.9)	0.0	4.6 (2.6)	6.1 (3.8)	12.0 (2.6)	12.9 (1.3)	
Shift	1.1 (1.2)	3.8 (1.5)	1.7 (1.3)	2.2 (0.9)	3.7 (1.7)	0.3 (0.3)	2.4 (2.4)	8.1 (6.1)	1.2 (0.6)	2.4 (0.5)	
Others	0.0	2.5 (1.0)	0.7 (0.5)	3.1 (1.2)	3.0 (1.4)	0.7 (0.6)	0.0	11.6 (9.0)	1.0 (0.5)	2.0 (0.4)	
BMI category											0.009
Normal/underweight	82.8 (11.7)	82.9 (2.5)	79.7 (3.2)	72.2 (3.2)	68.5 (4.0)	66.8 (4.4)	69.4 (8.0)	70.3 (9.6)	70.9 (2.6)	74.2 (1.3)	
Overweight	17.2 (11.7)	14.5 (2.2)	15.0 (2.6)	22.5 (3.0)	27.6 (4.0)	26.4 (3.3)	30.5 (8.0)	17.3 (7.8)	24.4 (2.7)	21.2 (1.1)	
Obese	0.0 (0.0)	2.6 (1.2)	5.2 (1.9)	5.3 (1.8)	3.9 (1.5)	6.8 (1.9)	0.1 (0.1)	12.4 (7.0)	4.7 (1.3)	4.5 (0.7)	

*Estimated mean (standard error).

The prevalence estimates of the metabolic syndrome by demographic characteristics stratified by gender are shown in Table 3. Among the male workers, the subgroup with the highest prevalence of the metabolic syndrome was the obese group (68.3%), followed by the overweight group (39.1%) and the group engaging in high-risk drinking everyday (38.1%). In the female workers, the group of those aged ≥ 70 years (53.5%) showed the highest prevalence of the metabolic syndrome, followed by the obese group (46.1%).

**Table 3:** The prevalence of the metabolic syndrome according to demographic characteristics

	**Male**		**Female**	
**Demographics**	Number ^*†^	**Prevalence (95% confidence intervals)**^**‡**^	Number^*†^	**Prevalence (95% confidence intervals)**^**‡**^
Age group				
20–29 years	152/1,942,533	9.2 (4.1–14.3)	231/1,733,502	3.5 (0.7–6.2)
30–39 years	428/3,276,013	17.8 (13.5–22.1)	291/1,634,571	5.5 (2.0–9.0)
40–49 years	477/3,659,709	25.9 (21.3–30.4)	371/2,433,142	12.5 (8.2–16.8)
50–59 years	392/2,681,787	35.7 (30.2–41.2)	374/1,891,022	30.8 (25.2–36.5)
60–69 years	297/1,176,249	36.4 (29.5–43.3)	188/758,966	38.0 (30.1–45.8)
≥ 70 years	113/415,885	17.4 (9.1–25.8)	93/373,407	53.5 (43.5–63.4)
Education				
≤ Elementary school	262/1,377,314	28.7 (21.5–35.8)	407/1,912,522	40.9 (35.6–46.2)
Middle school	218/1,385,292	35.9 (29.0–42.8)	175/989,224	24.4 (16.6–32.3)
High school	637/5,078,713	20.1 (16.4–23.7)	491/3,089796	9.3 (6.1–12.5)
≥ University	742/5,310,859	23.6 (19.9–27.4)	481/2,833,069	7.6 (4.2–11.1)
Household income				
Low	224/1,362,396	25.9 (18.9–32.9)	239/1,171,008	35.3(28.6–41.9)
Low–middle	476/3,488,782	21.4 (16.9–25.8)	366/2,177,822	19.8 (15.3–24.2)
Middle–high	568/4,173,072	23.8 (19.4–28.2)	448/2,712,764	14.3 (10.7–18.0)
High	575/4,051,739	25.5 (21.1–29.9)	481/2,648,160	10.4 (7.1–13.7)
High–risk drinking				
Never	275/1,681,687	14.7 (9.6–19.9)	473/2,633,998	15.4 (11.2–19.7)
Once a month	644/4,713,082	21.2 (17.4–24.9)	494/2,956,125	12.8 (9.3–16.4)
Once a weak	490/3,727,662	26.0 (21.6–30.4)	130/932,867	17.4 (9.6–25.2)
Everyday	226/1,602,909	38.1 (30.7–45.5)	23/152,018	31.1 (8.8–53.4)
Smoking status				
Non–smoker	320/2,194,133	19.5 (14.4–24.5)	1,394/7,721,944	17.5 (14.9–20.2)
Ex–smoker	697/4,369,598	27.0 (22.9–31.0)	81/546,406	16.0 (7.0–25.0)
Current smoker	841/6,585,245	23.7 (20.1–27.3)	78/547,078	15.8 (6.7–24.9)
Vigorous physical activity				
None	1,035/6,872,680	25.0 (21.9–28.1)	1,144/6,444,936	17.8 (14.4–20.7)
< 5 days per week	659/4,976,027	23.7 (19.5–28.0)	293/1,726,743	17.3 (12.5–22.2)
≥ 5 days per week	164/1,300,269	20.2 (12.7–27.8)	115/633,191	14.8 (7.9–21.7)
Work hours				
≤ 40 hours per week	666/4,555,070	22.5 (18.6–26.5)	819/4,514,730	17.3 (13.9–20.7)
> 40 hours per week	1,193/8,597,108	24.9 (21.8–28.0)	735/4,309,882	17.3 (14.3–20.4)
Work schedule pattern				
Day working	1,526/10,805,797	24.5 (21.9–271)	1,312/7,305,852	18.3 (15.4–21.2)
Non–day working	328/2,319,154	22.0 (16.6–27.4)	238/1,501,207	12.4 (6.8–18.1)
BMI category				
Underweight	40/272,239	0.0 (0.0–0.0)	85/539,963	0.0 (0.0–0.0)
Normal	1,099/7,673,502	12.9 (10.3–15.4)	1,053/6,039,293	9.4 (7.5–11.4)
Overweight	654/4,672,170	39.1 (34.3–43.9)	350/1,858,859	41.9 (35.7–48.1)
Obese	61/500,005	68.3 (54.1–82.5)	65/384,483	46.1 (32.3–59.9)

^*^Subject number/estimated population number. ^†^Subject number varies due to item which was not answered. ^‡^Unadjusted prevalence of the metabolic syndrome (95% confidence intervals).

Unadjusted and age-adjusted prevalence estimates for each occupational group stratified by gender are presented in Table 4. In the male workers, the overall unadjusted and age-adjusted prevalence estimates of the metabolic syndrome were 24.1% (95% CI: 21.7-26.5%) and 23.2% (95% CI: 21.0-25.5%), respectively. The prevalence estimates in male non-manual workers were 26.3% (95% CI: 22.3-30.2%) and 26.4% (95% CI: 22.3-30.5%), respectively, after age adjustment, which were the highest among the three occupational groups. In the occupational subgroups, “equipment, machine operation and assembling” workers showed the highest unadjusted prevalence of the metabolic syndrome (35.4%, 95% CI: 28.4-42.4%), followed by “managers” (31.7%, 95% CI: 18.1-45.3%) and “clerks” (26.9%, 95% CI: 21.0-32.8%). The highest age-adjusted prevalence for male workers was found in “professional and related” workers (27.3%, 95% CI: 20.9-33.6%), followed by “equipment, machine operation and assembling” workers (26.5%, 95% CI: 21.1-32.0%) and “managers” (26.5%, 95% CI: 14.2-38.8%).

**Table 4:** Unadjusted and age-adjusted prevalence of the metabolic syndrome by category of the Korean Standard Classification of Occupations stratified by gender

		**Prevalence of the metabolic syndrome** ** (95% confidence intervals)**	
**Occupational groups**	Number^*^	**Unadjusted**	**Age-adjusted**
Overall male workers	1,859/13,152,179	24.1 (21.7–26.5)	23.2 (21.0–25.5)
Non-manual workers	676/4,905,005	26.3 (22.3–30.2) ^†^	26.4 (22.3–30.5) ^†^
Managers	61/362,106	31.7 (18.1–45.3) ^†^	26.5 (14.2–38.8) ^†^
Professional and related workers	335/2,531,917	25.0 (19.3–30.8) ^†^	27.3 (20.9–33.6) ^†^
Clerks	280/2,010,981	26.9 (21.0–32.8) ^†^	24.6 (18.1–31.1)
Service/sales workers	304/2,302,276	19.9 (14.6–25.2)	21.5 (15.9–27.1)
Service workers	104/910,156	15.7 (8.1–23.3)	17.5 (9.0–26.0)
Sales workers	200/1,392,120	22.7 (15.3–30.0)	23.0 (16.2–29.8)
Manual workers	879/5,944,897	23.9 (20.6–27.1)	20.1 (17.3–23.0)
Skilled agricultural, forestry and fishery workers	272/1,589,656	24.3 (18.2–30.4)	15.6 (10.9–20.2)
Craft and related trades workers	206/1,733,288	15.7 (10.6–20.7)	16.4 (11.0–21.9)
Equipment, machine operating and assembling workers	240/1,597,780	35.4 (28.4–42.4) ^†^	26.5 (21.1–32.0) ^†^
Elementary workers	161/1,024,171	19.2 (11.3–27.2)	16.9 (9.4–24.4)
Overall female workers	1,544/8,824,612	17.3 (14.8–19.8)	19.5 (17.3–21.6)
Non-manual workers	545/3,325,220	7.7 (4.4–10.9)	20.8 (11.3–30.2) ^†^
Managers	8/22,859	12.8 (−9.1–34.8)	10.6 (−6.7–27.9)
Professional and related workers	334/1,998,628	7.3 (3.5–11.2)	27.9 (21.5–34.3) ^†^
Clerks	203/1,303,732	8.1 (2.6–13.5)	9.8 (2.7–16.9)
Service/sales workers	428/2,502,853	16.9 (12.8–20.9)	18.4 (13–22.9)
Service workers	215/1,340,314	16.9 (11.4–22.3)	20.5 (14.5–26.6) ^†^
Sales workers	213/1,162,539	16.9 (11.2–22.5)	17.6 (11.9–23.2)
Manual workers	581/2,996,538	28.4 (23.9–32.9) ^†^	20.8 (16.9–24.7) ^†^
Skilled agricultural, forestry and fishery workers	225/1,011,474	39.2 (31.8–46.6) ^†^	22.0 (15.2–28.9) ^†^
Craft and related trades workers	42/258,235	19.1 (4.4–33.8)	12.0 (2.8–21.2)
Equipment, machine operating and assembling workers	35/158,745	7.8 (−2.5–18.1)	6.2 (−1.7–14.2)
Elementary workers	279/1,568,083	25.0 (19.1–31.0) ^†^	20.0 (14.5–25.5) ^†^

^*^Subject number/estimated population number. ^†^The prevalence estimate for each occupational group was above the upper bound of the 95% confidence interval for the overall prevalence estimate.

In the female workers, the estimates of overall unadjusted and age-adjusted prevalence of the metabolic syndrome were 17.3% (95% CI: 14.8-19.8%) and 19.5% (95% CI: 17.3-21.6%), respectively. Among the female workers, the age-adjusted prevalence of the metabolic syndrome was similar in both non-manual (20.8%, 95% CI: 11.3-30.2%) and manual (20.8%, 95% CI: 16.9-24.7%) workers, while the unadjusted prevalence was highest in manual workers (28.4%, 95% CI: 23.9-32.9%) and was lowest in non-manual workers (7.7%, 95% CI: 4.4-10.9%). In the individual occupational groups, the highest unadjusted prevalence was documented in “skilled agricultural, forestry and fishery” workers (39.2%, 95% CI: 31.8-46.6%), followed by “elementary” workers (25.0%, 95% CI: 19.1-31.0%) and “craft and related trades” workers (19.1%, 95% CI: 4.4-33.8%). “Professional and related” workers showed the highest age-adjusted prevalence of the metabolic syndrome among the female workers (27.9%, 95% CI: 21.5-34.3%), followed by “skilled agricultural, forestry and fishery” workers (22.0%, 95% CI: 15.2-28.9).

Table 5 shows the age-adjusted prevalence of the individual components of the metabolic syndrome by occupational group. Among the three occupational groups of non-manual, service/sales, and manual workers, the non-manual workers had the highest prevalence of high blood pressure (40.1%), hypertriglyceridemia (39.4%), low HDL-cholesterol level (20.5%), and hyperglycemia (31.8%) among the male workers. The highest prevalence of abdominal obesity was found in the service/sales workers among the male workers (28.6%). In the occupational subgroups, the prevalence estimate of abdominal obesity for male workers was highest in the “managers” (35.4%), followed by “craft and related trades” workers (31.1%) and “equipment, machine operation and assembling” workers (31.1%). “Clerks” also showed the highest prevalence of high blood pressure (51.6%) and hyperglycemia (34.5%) for male workers. Among the male workers, the highest prevalence of hypertriglyceridemia was found in the “equipment, machine operation and assembling” workers (44.9%), and the highest prevalence of low HDL-cholesterol levels was identified in the “professional and related” workers (22.0%), followed by “equipment, machine operation and assembling” workers (19.5%). Unlike the male workers, the prevalence of abdominal obesity among the female workers was highest in the “skilled agricultural, forestry and fishery” workers (50.5%), followed by “service” workers (42.0%), “professional and related” workers (41.8%), and “elementary” workers (40.9%). The highest prevalence of high blood pressure among the female workers was documented in the “craft and related trades” workers (32.3%). The highest prevalence of hypertriglyceridemia was found in the “managers” (31.8%). In the female workers, the “skilled agricultural, forestry and fishery” workers (43.0%) and “professional and related” workers (43.0%) showed the highest prevalence of low HDL-cholesterol levels. However, the prevalence of low HDL-cholesterol levels among male workers was lowest in the “skilled agricultural, forestry and fishery” workers (9.0%). The prevalence of hyperglycemia among the female workers was highest in the “professional and related” workers (24.7%).

**Table 5:** Age-adjusted prevalence of the components of the metabolic syndrome by category of the Korean Standard Classification of Occupations

	**Non-manual workers**			**Service/sales workers**		**Manual workers**					
**Components**	**Managers**	**Professional and related workers**	**Clerks**	**Service workers**	**Sales workers**	**Skilled agricultural, forestry and fishery workers**	**Craft and related trades workers**	**Equipment, machine operation and assembling workers**	**Elementary workers**	**Total**	**p-value**
Male workers											
WC ≥ 90 cm (M)/80 cm (W)		24.5 (2.5)		28.6 (3.5)			24.0 (1.9)			25.1 (1.3)	0.946
	35.4 (8.7)	21.1 (2.8)	25.7 (3.7)	25.7 (4.7)	28.2 (3.9)	22.8 (3.5)	31.1 (4.3)	31.0 (3.2)	18.2 (4.2)		0.705
BP ≥ 130/85 mmHg or treated		40.1 (2.5)		33.3 (3.3)			32.7 (1.7)			36.3 (1.4)	0.036
	46.0 (6.9)	33.2 (3.5)	51.6 (2.6)	29.8 (4.4)	35.5 (3.8)	26.6 (3.3)	29.0 (3.5)	32.7 (2.6)	36.2 (4.1)		<0.001
TG ≥ 150 mg/dl		39.4 (2.6)		35.5 (2.7)			35.3 (2.2)			37.4 (1.4)	0.225
	41.8 (8.0)	43.9 (3.3)	33.7 (3.8)	35.2 (5.0)	37.7 (3.3)	25.9 (4.6)	34.7 (4.0)	44.9 (2.9)	29.9 (4.9)		0.119
HDL-C < 40 mg/dl		20.5 (2.2)		13.3 (2.5)			18.3 (1.8)			19.2 (1.2)	0.024
	14.9 (4.1)	22.0 (3.2)	16.9 (2.5)	12.6 (3.8)	13.5 (3.0)	9.0 (1.5)	17.1 (3.0)	19.5 (2.9)	18.8 (4.6)		0.119
FPG ≥ 100 mg/dl or treated		31.8 (2.5)		29.3 (3.4)			26.2 (1.7)			28.6 (1.3)	0.191
	31.2 (7.6)	31.8 (3.2)	34.5 (4.1)	26.2 (5.1)	29.2 (3.8)	20.3 (2.4)	25.8 (3.5)	27.1 (2.8)	26.8 (3.4)		0.284
Female workers											
WC ≥ 90 cm (M)/80 cm (W)		34.6 (4.8)		39.0 (2.5)			41.8 (3.3)			36.8 (1.4)	<0.001
	19.5 (12.2)	41.8 (3.4)	24.6 (4.3)	42.0 (3.4)	36.9 (3.2)	50.5 (6.7)	32.0 (5.1)	22.4 (5.4)	40.9 (5.0)		<0.001
BP ≥ 130/85 mmHg or treated		21.0 (1.8)		23.8 (2.2)			27.6 (1.9)			25.6 (1.2)	<0.001
	29.4 (10.8)	19.0 (2.2)	23.2 (3.3)	26.4 (2.9)	21.3 (3.0)	29.0 (4.2)	32.3 (4.8)	7.1 (4.0)	27.0 (2.5)		<0.001
TG ≥ 150 mg/dl		15.7 (2.7)		23.3 (2.9)			17.6 (2.1)			17.9 (1.1)	<0.001
	31.8 (13.2)	18.4 (2.1)	5.6 (2.0)	22.7 (4.5)	23.6 (3.4)	16.1 (2.6)	9.5 (4.0)	8.3 (4.8)	18.4 (2.8)		<0.001
HDL-C < 50 mg/dl		34.9 (4.7)		32.2 (2.9)			34.7 (3.1)			32.8 (1.4)	<0.001
	15.1 (10.8)	43.0 (2.6)	21.2 (4.3)	33.2 (3.6)	33.0 (4.0)	43.0 (5.8)	37.9 (8.4)	16.8 (5.1)	32.1 (5.1)		<0.001
FPG ≥ 100 mg/dl or treated		19.8 (4.5)		16.1 (1.9)			19.0 (2.0)			18.1 (1.1)	<0.001
	9.4 (8.6)	24.7 (3.7)	15.6 (3.5)	15.3 (3.1)	17.1 (2.8)	14.9 (1.8)	9.0 (2.2)	15.2 (5.8)	20.7 (2.5)		<0.001

Abbreviation: All values were the estimated percentage (standard error). *WC* waist circumference, *BP* blood pressure, *TG* triglyceride, *HDL*-*C* high-density lipoprotein cholesterol, *FPG* fasting plasma glucose, *M* male, *F* female.

Table 6 shows the results of multiple logistic regression analyses. Manual workers were significantly less likely to have the metabolic syndrome (odds ratio: 0.62, 95% CI: 1.19-5.86) relative to non-manual workers among male workers after adjustment for age, level of education, household income, high risk drinking, smoking status, and vigorous physical activity (Model I). After additional adjustment for work hours and work schedule pattern, both manual (0.63, 95% CI: 0.39-0.99) and service/sales (0.59, 95% CI: 0.41-0.85) workers had significantly lower odds of having the metabolic syndrome compared to non-manual workers among the male workers (Model II). There were no significant associations between the metabolic syndrome and the occupational group among female workers in either model. When comparing the subgroups of service/sales and manual workers with the non-manual workers as a reference, “service” workers, “craft and related trades” workers, and “elementary” workers showed significantly lower odds among the male workers.

**Table 6:** Multiple logistic regression to assess the relationship between occupation and meeting criteria for the metabolic syndrome among Korean workers aged ≥20 years

	**Odds ratio (95% confidence intervals)**	
	**Male**	**Female**
Model I^*^		
Occupational group		
Non-manual workers	1.00 (reference)	1.00 (reference)
Service/sales workers	0.66 (0.42–1.02)	0.98 (0.45–2.12)
Service workers	0.49 (0.25–0.96)	0.92 (0.36–2.32)
Sales workers	0.74 (0.43–1.28)	1.12 (0.51–2.46)
Manual workers	0.62 (0.44–0.88)	1.00 (0.46–2.16)
Skilled agricultural, forestry and fishery workers	0.38 (0.20–0.70)	1.56 (0.56–4.33)
Craft and related trades workers	0.39 (0.23–0.66)	0.78 (0.21–2.83)
Equipment, machine operating and assembling workers	1.22 (0.82–1.83)	0.88 (0.16–4.67)
Elementary workers	0.43 (0.21–0.91)	0.84 (0.38–1.86)
Model II^†^		
Occupational groups		
Non-manual workers	1.00 (reference)	1.00 (reference)
Service/sales workers	0.63 (0.39–0.99)	1.04 (0.48–2.25)
Service workers	0.45 (0.22–0.93)	0.98 (0.39–2.47)
Sales workers	0.73 (0.42–1.26)	1.16 (0.53–2.57)
Manual workers	0.59 (0.41–0.85)	1.02 (0.47–2.21)
Skilled agricultural, forestry and fishery workers	0.37 (0.20–0.69)	1.54 (0.56–4.25)
Craft and related trades workers	0.37 (0.22–0.63)	0.78 (0.22–2.81)
Equipment, machine operating and assembling workers	1.16 (0.77–1.75)	0.90 (0.17–4.87)
Elementary workers	0.42 (0.19–0.91)	0.87 (0.39–1.94)

* Model I: Adjusted for age, level of education, household income, high-risk drinking, smoking status, and vigorous physical activity.

^†^ Model II: Adjusted for Model I factors, plus work hours, and work schedule.

## Discussion

In this study, we assessed the prevalence of the metabolic syndrome by occupational group among Korean workers and evaluated the risk of the metabolic syndrome among each of the occupational groups. Our study demonstrates variability in the prevalence of the metabolic syndrome by occupational group, and found the greatest risk for the metabolic syndrome in male non-manual workers.

Several studies have identified differences in the prevalence of the metabolic syndrome by occupational group [8-10]. In Spanish workers, the presence of the metabolic syndrome after age adjustment was greatest in the “machine installers, operators, and assemblers” (15.1%) group among males, with an overall prevalence of 9.5% (11.6% in male workers and 4.1% in female workers). Manual workers had a higher prevalence than non-manual workers in both males and females [8]. According to a study of the U.S. working population, the overall prevalence of the metabolic syndrome was 20.6% (20.2% in male workers and 21.4% in female workers) with the greatest unadjusted prevalence among “transportation and material occupations” (33.1%) and the greatest age-adjusted prevalence among “food preparation and food service workers” (31.1%) [9]. In a study using the KNHANES (2005) data, the prevalence of the metabolic syndrome among Korean workers was found to be 21.8% (22.5% in male worker and 15.9% in female), and the prevalence was higher in manual workers than in non-manual workers [10].

In this study, non-manual workers showed higher unadjusted and age-adjusted prevalences of the metabolic syndrome than service/sales and manual workers among the male workers. Most of the workers in the non-manual groups spend most of their work hours sedentary [15]. Furthermore, they may also engage in less physical activity relative to other workers. Actually, the proportion of those engaging in vigorous physical activity at least 5 days a week in these groups was lower than in the other workers in this study.

In the male non-manual group, the “managers” showed the highest prevalence estimate (31.7%), but the prevalence in the “equipment, machine operating and assembling” workers was the highest (35.4%) among the nine occupational subgroups before age adjustment. On the other hand, after age adjustment, the highest prevalence was observed in “professional and related” workers (27.3%), followed by “managers” (26.5%) and “equipment, machine operating and assembling” workers (26.5%). The subgroups of non-manual workers including “managers”, “professional and related” workers, and “clerks” had a higher level of education and household income than the manual working groups in both the male and female workers. Several studies have shown an inverse association between socioeconomic status and the metabolic syndrome in women, but no association in men [16-18]. However, a recent study of the Korean population using 2007–2008 KNHANES data has reported that socioeconomic status (SES) had a positive association with the metabolic syndrome for men and an inverse association for women [19]. The researchers suggested differences in health behaviors including smoking and drinking, food consumption, heath care assessment, and psychological stress according to SES as possible explanations for the inverse association in women. The authors explained that the finding of high prevalence of metabolic syndrome in men with highest household income was consistent with an earlier study using data from the 1998 to 2001 KNHANES, which showed a positive relationship between obesity and higher SES in men [20]. This positive association among men might be applied to explain the data on the male workers in our study. The subgroups of non-manual male workers including “managers”, “professional and related” workers, and “clerks” had a higher proportion of overweight and obese individuals than the other occupational groups in our study.

Among the subgroups of male manual workers, only “equipment, machine operating and assembling” workers showed a significantly higher prevalence of the metabolic syndrome than the prevalence for the male workers overall before and after age adjustment, and the prevalence was similar to the prevalence in non-manual workers. This observation was in concordance with a previous study in Spain, which showed the highest prevalence of the metabolic syndrome in the “machine installers, operators and assemblers” group among all of the occupational groups [8]. When the demographic characteristics of this group were considered, they were found to have a higher level of education (high school education and more) and household income (middle to high) in the manual worker groups. It seems that higher socioeconomic status, among other factors, might have influenced this result.

Among the female workers, although the unadjusted prevalence was greatest in the manual workers (28.4%) and lowest in the non-manual workers (7.3%), this difference disappeared after age standardization (20.8% versus 20.8%, respectively). Among the subgroups of the female non-manual workers, “professional and related” workers showed the lowest unadjusted prevalence of the metabolic syndrome (7.3%) and the highest age-adjusted prevalence (27.9%). This was the main cause for the increase in the overall prevalence in non-manual workers after age adjustment. Although not presented in this paper, the age distribution of female “professional and related” workers was skewed toward younger ages. At this age group, the prevalence of the metabolic syndrome was lower, but compared to the younger group, the prevalence in the older age group was extremely high. In the multiple logistic regression analysis, there was no significant association found between occupational groups and the metabolic syndrome in female workers.

Unlike the prevalence estimate of the male “skilled agricultural, forestry and fishery” workers, which was significantly lower than the overall male prevalence after age adjustment, female workers of the same occupational group continued to show significantly higher prevalence of the metabolic syndrome compared to the overall prevalence after age adjustment. This distinct feature was also evident in the analysis of individual components of the metabolic syndrome. Female “skilled agricultural, forestry and fishery” workers presented the highest age-adjusted prevalence of abdominal obesity and low HDL-cholesterol levels among the occupational subgroups. Conversely, male “skilled agricultural, forestry and fishery” workers showed the lowest age-adjusted prevalence of low HDL-cholesterol levels and a relatively lower prevalence of abdominal obesity. These gender differences may come from post-menopausal hormonal changes in female workers, given that recent studies have shown that the prevalence of the metabolic syndrome was higher in postmenopausal women than in premenopausal women [21-24]. In the present study, “skilled agricultural, forestry and fishery” workers were the most aged group; that is, more female workers of that group could be expected to be post-menopausal based on their age than in the other groups.

We categorized service and sales workers into a separate group, apart from the non-manual and manual workers. Our results showed that service/sales workers had characteristics intermediate to the other two occupational groups with regard to SES. Likewise, the age-adjusted prevalence of the metabolic syndrome among the male service/sales workers was between the prevalence in non-manual workers and the prevalence in manual workers. In the women also, considering that the dramatic increase of the prevalence in non-manual workers after age adjustment was mainly due to the uneven distribution of age, the prevalence of the metabolic syndrome in service/sales workers stood between those of manual and non-manual workers.

In this study, the work schedule pattern was included as a covariate. In both male and female workers, the percentages of night/overnight or shift work were the highest for the service workers. Generally, it is known that shift work causes the disturbance of sleep and normal circadian rhythms, and it may increase psychosocial stress, predisposing the worker to physiological disturbances related to the metabolic syndrome and cardiovascular disease [25,26]. Recent studies have shown that shift work is closely related to the increased risk of the metabolic syndrome [27-31]. However, the prevalence of the metabolic syndrome in this group was not higher compared to the other occupational groups. In addition, the result of multiple logistic regression analysis did not show a significant association between the metabolic syndrome and work schedule (data not shown).

The strengths of our investigation are the use of a large sample representative of the Korean population and the analysis of the prevalence of the metabolic syndrome in 9 occupational groups considering multiple variables such as age, gender, level of education, household income, and smoking status. The limitations are an inability to draw causal inferences due to the cross-sectional design and unavailability of detailed information about work-related condition in the KNHANES.

In conclusion, our study demonstrated variability in the prevalence of the metabolic syndrome by occupational group among Korean workers. In the male workers, non-manual workers appeared to be more vulnerable to the metabolic syndrome compared to service/sales workers or manual workers, but not in the female workers. Future research should evaluate factors that may influence the occurrence of the metabolic syndrome in each occupational group so that our findings can be utilized to establish appropriate preventive measures for the metabolic syndrome and co-occurring diseases including cardiovascular disease and diabetes.

## Competing interests

The authors declare that they have no competing interests.

## Authors’ contributions

DH Kim and JY Ryu designed the research. S Hong and CH Kim collected the data. JY Ryu and S Lee performed the statistical analysis. JY Ryu, JT Lee, JH Kim, and DH Kim interpreted the data. JY Ryu and S Lee wrote the manuscript. All of the authors read and approved the final manuscript.
